# Synthesis and Trapping of the Elusive *Ortho*-Iminoquinone Methide Derived from α-Tocopheramine and Comparison to the Case of α-Tocopherol

**DOI:** 10.3390/molecules30153257

**Published:** 2025-08-04

**Authors:** Anjan Patel, Thomas Rosenau

**Affiliations:** Department of Natural Sciences and Sustainable Resources, Institute of Chemistry of Renewable Resources, BOKU University, Konrad-Lorenz-Straße 24, 3430 Tulln, Austria

**Keywords:** tocopheramine, vitamin E, oxidation, *ortho*-iminoquinone methide, hetero-Diels–Alder reaction with inverse electron demand, spiro-dimerization

## Abstract

Tocopheramines are a class of antioxidants which are distinguished from tocopherols (vitamin E) by the presence of an amino group instead of the phenolic hydroxyl group. α-Tocopheramine is intensively studied for biomedical applications but also as a stabilizer for synthetic and natural polymers, in particular for cellulose solutions and spinning dopes for cellulosic fibers. This study addresses a fundamental difference in the oxidation chemistry of α-tocopheramine and its tocopherol counterpart: while the formation of the ortho-quinone methide (o-QM) involving C-5a is one of the most fundamental reactions of α-tocopherol, the corresponding ortho-iminoquinone methide (o-IQM) derived from α-tocopheramine has been elusive so far. Synthesis of the transient intermediate succeeded initially via 5a-hydroxy-α-tocopheramine, and its occurrence was confirmed by dimerization to the corresponding spiro-dimer and by trapping with ethyl vinyl ether. Eventually, suitable oxidation conditions were found which allowed for the generation of the o-IQM directly from α-tocopheramine. The underlying oxidation chemistry of α-tocopherol and α-tocopheramine is concisely discussed.

## 1. Introduction

**Scheme 1:**
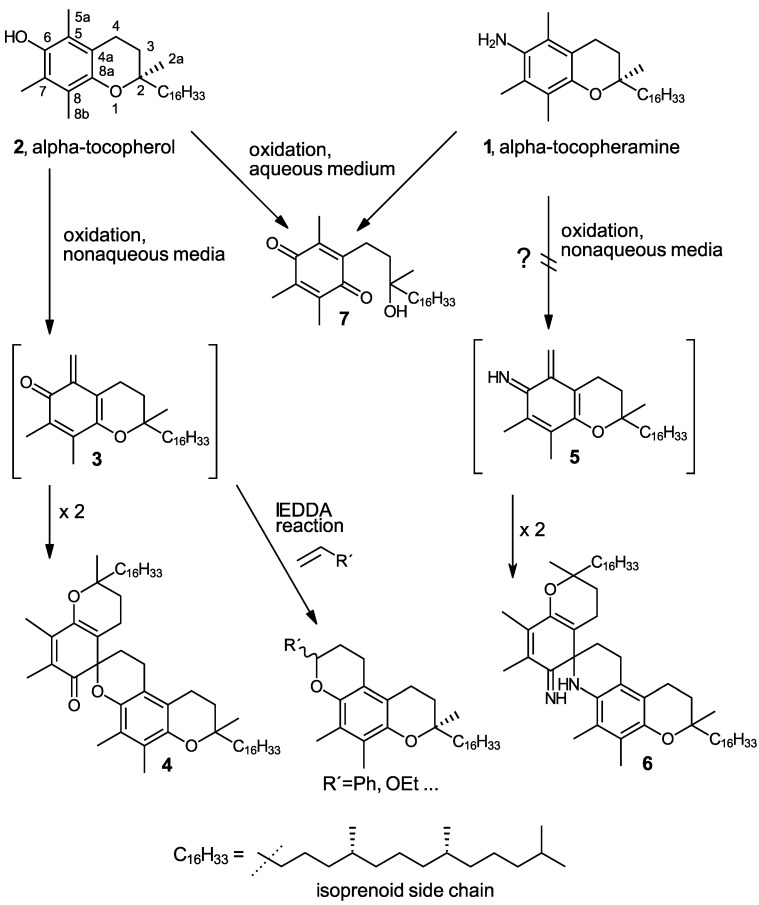
Chemical structures of the parent compounds α-tocopheramine (**1**), α-tocopherol (**2**), and a comparison of the chemistry of their oxidation as well as trapping and dimerization of their ortho-quinoid oxidation intermediates. The question mark indicates the elusive reaction path.

First reported in 1942, α-tocopheramine (**1**) has long been a niche product in the shadow of its “big brother” α-tocopherol (**2**), the main component in vitamin E and the most important lipophilic antioxidant in vivo [1]. Replacement of the phenolic OH group in tocopherols affords the corresponding tocopheramines [2,3], as shown in Scheme 1. Tocopheramines possess similar bioactivities [2,4], even exceeding those of tocopherols in some cases [5,6]. Early proposals for larger-scale use did not prevail, such as utilization as food [7] and feed additives [8], surfactants [9], and polymer stabilizers [10]. Recently, α-tocopheramine has been used as a stabilizer for cellulose solutions in *N*-methylmorpholine-*N*-oxide monohydrate, acting against cellulose degradation and solvent decomposition [11,12], as a possible alternative to the commonly employed stabilizer propyl gallate. Also, α-tocopheramine (**1**) and its *N*-methyl derivative (**1a**) have shown very good properties as stabilizers of cellulose in 1,3-dialkylimidazolium ionic liquid (IL) solvents upon spinning cellulosic specialty fibers [13]. An appropriate stabilizer in this application field is supposed to simultaneously curb cellulose degradation, solvent decomposition, and the formation of chromophores, which impair both solvent recycling and fiber quality. In contrast to other natural antioxidants, such as α-tocopherol and its derivatives, and synthetic antioxidants, such as propyl gallate, butylated hydroxyanisol (BHA), or butylated hydro-xytoluene (BHT), which performed well in one or two of these tasks, α-tocopheramine was superior in all three. It was thus only logical that the underlying chemistry, side reactions, and byproduct formation of tocopheramines have experienced a surge in interest recently.

A particularly obvious difference in the oxidation chemistries of α-tocopheramine (**1**) and its phenolic counterpart α-tocopherol (**2**) is the almost ubiquitous occurrence of the ortho-quinone methide (o-QM) **3** derived from α-tocopherol [14,15] as a central intermediate as well as its dimerization product, the α-tocopherol spiro-dimer (**4**) [16,17] (see Scheme 1), while the corresponding α-tocopheramine-derived structures, i.e., the ortho-iminoquinone methide (o-IQM) **5** derived from α-tocopheramine and its spiro-dimer (**6**), are not formed (with the exception of traces of the latter found in IL/cellulose spinning dopes). Non-aqueous media and oxidation conditions that usually provide quantitative yields of o-QM intermediate **3** and spiro-dimer **4** from α-tocopherol afforded only *N*-oxidized products in the case of α-tocopheramine as the starting material, but there were no quinoid products whatsoever. In aqueous media, no differences in reaction behavior are observed, and both α-tocopheramine and α-tocopherol form para-tocopherylquinone (**7**) [18,19] quantitatively, as shown in Scheme 1. As a consequence, trapping the ortho-quinoid structures in Diels–Alder processes—more specifically, in hetero-Diels–Alder reactions with inverse electron demand (IEDDA reactions)—which were intensively used for the tocopherol-derived o-QM (**3**) to prepare novel tocopherol derivatives—have not yet been performed analogously in α-tocopheramine chemistry, since the ortho-iminoquinone methide 5 itself has been elusive so far.

In this study, we would like to communicate the preparation of the o-IQM intermediate **5** via a “detour”—the elimination of water from either the 5a-hydroxy derivative of α-tocopheramine (**10**) or the N-hydroxy derivative α-tocopherhydroxylamine (**12**). This path was necessary because the direct oxidation from α-tocopheramine (**1**) to o-IQM **5** did not appear to be viable (see Scheme 1) until—admittedly by serendipity—suitable oxidation conditions were found. These beneficial conditions eventually allowed for the investigation and optimization of trapping reactions for **5** and the preparation of the corresponding trapping products and dimerization products as standard for analytical purposes. A possible direct formation of **5** from α-tocopheramine **1** in small amounts, as occurring in different cellulosic fiber-related applications, thus can now be analytically detected and followed much better than hitherto possible.

## 2. Results and Discussion

**Scheme 2:**
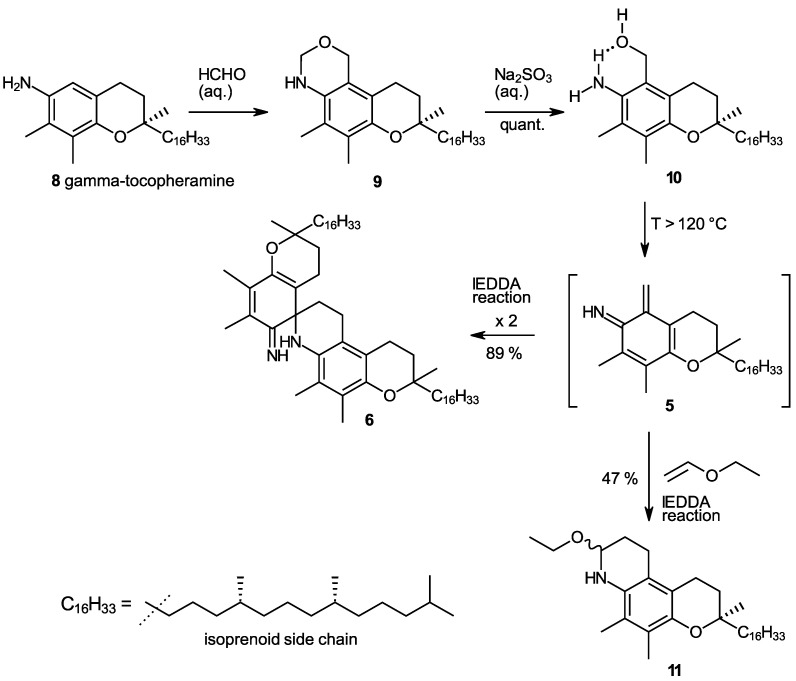
Synthesis of 5a-hydroxy-α-tocopheramine (**10**) and its use as a precursor of the ortho-iminoquinone intermediate **5** (o-IQM) which undergoes self-dimerization to spiro-dimer **6** or trapping reactions in hetero-Diels–Alder processes with inverse electron demand (IEDDA), forming heterotricyclic compound **11** in a reaction with ethyl vinyl ether.

In our studies on the *N*-demethylation of *N*,*N*-dimethyltocopheramines via the corresponding *N*-oxides (cf. the procedure in [20]), we observed a high propensity of γ-tocopheramine (**8**)—the tocopheramine derivative having a free, non-methylated 5-position—to form 5-hydroxymethylated or 5-aminomethylated byproducts. This guided us to study the reaction of γ-tocopheramine with aqueous formaldehyde, which proceeds neatly and quantitatively within 5 min simply by mixing the components. The initial product, however, is the tricyclic compound **9**, which by subsequent treatment with a concentrated sodium sulfite solution quantitatively provided 5a-hydroxy-α-tocopheramine (**10**) (Scheme 2). 5a-Hydroxy-α-tocopheramine (**10**) can also be conceived as the addition product of water to the elusive o-IQM (**5**) derived from α-tocopheramine. It was thus reasonable to expect that a reversal of this water addition process, i.e., the elimination of water from **5**, would be a possible way to produce o-IQM **5**. Indeed, refluxing compound **10** in ethyl vinyl ether—the “classic” trapping reagent for the o-QM **3** derived from α-toco-pherol—provided compound **11**, the trapping product of o-IQM **5**, in a yield of 47% (Scheme 2), which is a surprisingly high value considering that all previous attempts to trap and detect this o-IQM intermediate had failed. Besides trapping product **10**, 23% of α-tocopheramine (**7**), 8% of the spiro-dimer of α-tocopheramine (**6**), and unidentified, probably oligomeric, products were obtained in this reaction. This result was clear proof that the o-IQM intermediate **5** existed and that it can even be generated in reasonably high yields if the conditions are appropriate.

In a follow-up experiment, we heated 5a-hydroxy-α-tocopheramine (**10**) to 120 °C in solvents inert under the prevailing conditions (DMSO, o-dichlorobenzene, diphenyl ether) in the absence of trapping agents, which provided the dimerization product of o-IQM, the spiro-dimer of α-tocopheramine (**6**), in an excellent 89% yield for the solvent DMSO (85% and 91% in *o*-dichlorobenzene and diphenyl ether, respectively). Mechanistically, the elimination of water from **10** in a [1,4]-elimination produced the o-IQM intermediate **5** which, in turn, dimerized in a hetero-Diels–Alder process with inverse electron demand (IEDDA) [21,22] to spiro-dimer **6** by complete analogy to the well-known dimerization of the o-QM **3** derived from α-tocopherol into its spiro-dimer (**4**). This outcome further corroborated the intermediacy of o-IQM **5**. Its trapping in the presence of ethyl vinyl ether in a closed pressure reactor provided trapping product **11** in a 47% yield.

**Scheme 3:**
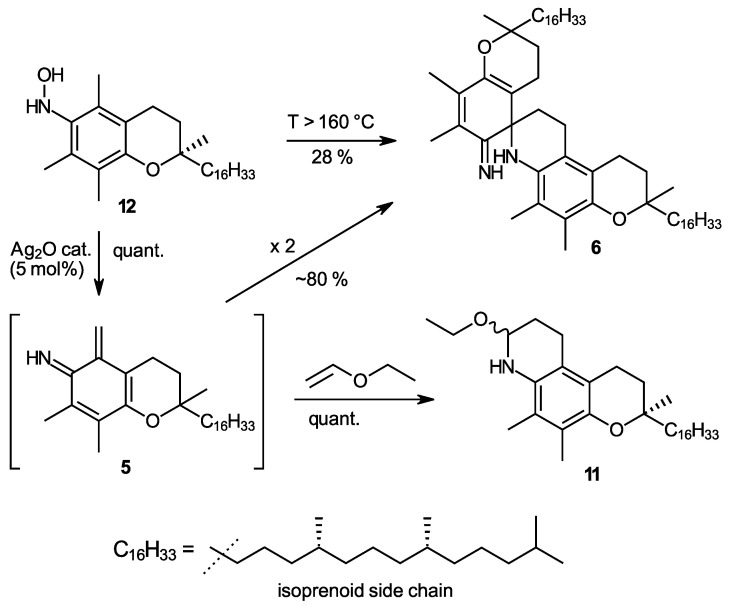
The synthesis of α-tocopherhydroxylamine (**12**) and its use as a precursor of the ortho-iminoquinone intermediate **5** (o-IQM). Treatment of **12** with catalytic amounts of freshly precipitated Ag_2_O at room temperature offers the first synthetically useful and quantitative access to the hitherto elusive o-IQM synthon, demonstrated exemplarily in a trapping reaction with ethyl vinyl ether.

In the above [1,4]-elimination mechanism, the released water molecule stems from the OH group at C-5a and a proton from the 6-amino group. We hypothesized that a similar elimination could also occur when the positions of OH and H are “swapped”, which would offer another approach to form the o-IQM intermediate. The starting material for such a reaction was α-tocopherhydroxylamine (**12**), a compound which can be obtained directly by oxidation from α-tocopheramine (**1**) [23]. Direct thermal elimination proceeded only at rather high temperatures, starting around 160 °C in diphenyl ether as the solvent. Although this reaction was accompanied by extensive byproduct formation, yields of spiro dimer **6** reached up to 28%, which was uninteresting as synthesis approached, yet it provided another indication of o-IQM intermediacy. Interestingly, freshly prepared silver oxide, one of the standard oxidants in tocopherol (vitamin E) chemistry, allowed for the cleavage of the N-O bond in hydroxylamine **12** and subsequent water elimination already at room temperature. Note the silver oxide in this reaction is not further oxidizing the hydroxylamine but rather enabling cleavage of the N-O bond, its role being that of a catalyst rather than that of an oxidant. This is also proven by the fact that the reaction required only 5 mol% of Ag_2_O to proceed quantitatively (Scheme 3). When the reaction was carried out in diethyl ether or dichloromethane, yields of spiro-dimer **6** were 78% and 83%, respectively, besides the formation of unidentified byproducts (mass spectrometry MS indicated the presence of trimeric compounds) and traces of α-tocopheryl quinone (**7**). In the presence of excess ethyl vinyl ether (10 molar equivalents), the formation of trapping product **11** was quantitative. Although in vitamin E chemistry, many quantitative or near-quantitative processes involving o-QM **3** are known; this reaction is the first example of quantitative generation of the analogous o-IQM **5**. The easy N-O cleavage in α-tocopherhydroxylamine (**12**) by silver oxide can now be used as straightforward and general access to the o-IQM (**5**) synthon and is the first approach to render this intermediate highly accessible. The ambient temperature of the generation rection, the requirement of only catalytic amounts of Ag_2_O, and the easy access to the starting hydroxylamine **12** are additional advantages.

**Scheme 4:**
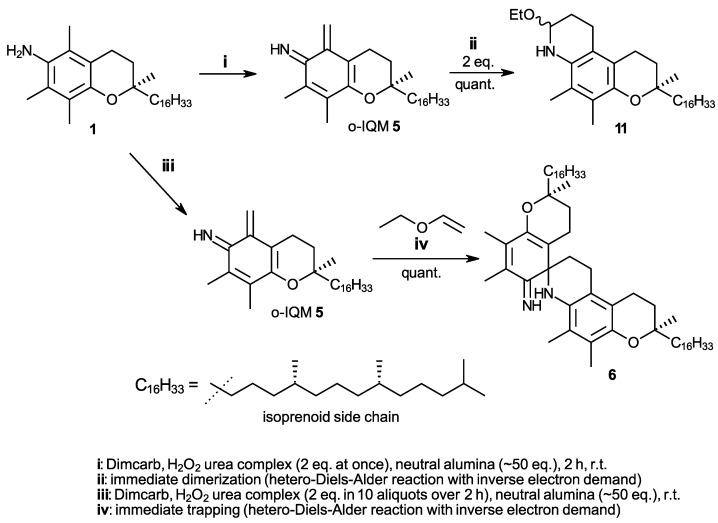
Synthesis of ortho-iminoquinone intermediate **5** (o-IQM) directly from α-tocopheramine (**1**) and its subsequent transformation by dimerization to spiro-dimer **6** or by trapping with ethyl vinyl ether to compound **11**.

In our previous work, to find a suitable straightforward synthesis for spiro-dimer **6** that would make gram amounts accessible, only the four oxidants DDQ, HOCl (from NMMO/AuCl_3_ (cat.) [24], Pb(OAc)_4_, or hydrogen peroxide (30% in water) gave promising results, albeit only when used in solvents with high ionicity. These were either DMAc containing the salt LiCl (8 wt%), a solvent known from the gel permeation chromatography of celluloses, or the dimcarb solvent, a liquid addition product of the two gases *N*,*N*-dimethylamine and carbon dioxide [25,26,27,28,29], which can be tracelessly removed at 60 °C by volatilization into the two gaseous components. However, α-tocopherylquinone (**7**) and *N*-oxidized products (nitro- and azo-derivative) [21,30] were always formed as byproducts. When hydrogen peroxide was applied as its solid urea adduct (2 eq.) rather than as an aqueous solution, the formation of *N*-oxidized products was completely avoided. Furthermore, the use of excess neutral alumina (50 eq., Brockmann grade 1 or 2) acting as an efficient drying agent ensured the immediate trapping of formed water and thus the reliable absence of α-tocopherylquinone [31]. The oxidation conditions in Scheme 4 thus provide the first quantitative approach to spiro-dimer **6** via o-IQM intermediate **5**.

The formation of the spiro-dimer **4** from α-tocopherol (**2**) proceeds neatly with quantitative or near-quantitative yields, inducible by many oxidants and proceeding in a large variety of aprotic solvents [15,16]. Also, the trapping reaction of the o-QM intermediate **3** with ethyl vinyl ether was very fast, with a reaction rate approx. 10 times faster than the dimerization [14]. A 10-fold excess of ethyl vinyl ether was usually sufficient to ensure near-quantitative formation of the trapping products and the absence of spiro-dimer **4** as a byproduct [32], with the oxidant to generate the o-QM intermediate **3** being added at once. These low-level requirements to generate and trap the o-QM intermediate in tocopherol chemistry—with regard to oxidants, solvents, and general reaction conditions—unfortunately do not find a parallel in tocopheramine chemistry. In fact, it is the contrary: when the o-IQM **5** generation was carried out starting from α-tocopheramine (**1**) with the oxidant H_2_O_2_–urea being added at once and an analogous trapping approach with a 10-fold excess of ethyl vinyl ether being used, only 34% of the trapping product **11** was obtained, besides the dominating spiro-dimer **6**. In the α-tocopheramine case, the trapping reaction was obviously not significantly faster than the dimerization, unlike the α-toco-pherol scenario. There were two general ways to promote the trapping process over dimerization, either by increasing the amount of ethyl vinyl ether being present or by decreasing the amount of generated o-IQM relative to the ethyl vinyl ether. After optimization of the reaction conditions, a 50-fold molar excess of vinyl ether, i.e., simply using the compound as a second solvent component, and the stepwise addition of the oxidant in 10 aliquots instead of one portion were chosen as appropriate trapping conditions. This setting reliably provided quantitative yields of trapping product **11**. The synthesis in Scheme 4 thus represents an alternative to the comparable, equally quantitative procedure in Scheme 3 which, however, uses a “detour” by requiring α-tocopherhydroxylamine (**12**) as the starting material. The significant advantage of the synthesis in Scheme 4 is that it starts directly from the easily accessible α-tocopheramine (**1**).

The generation reactions for o-IQM **5** in Scheme 2, Scheme 3 and Scheme 4 showed no dependence whatsoever on the atmosphere used. Although generally carried out under nitrogen, a test experiment in the presence of air did not provide lower yields of either spiro-dimer **6** or trapping product **11**. This indicated the absence of radical reactions for which an influence of atmospheric oxygen could be expected. Additionally, the reaction of α-tocopherhydroxylamine (**12**) to **11** via **5** was carried out in the presence of EMPO derivatives [33] as spin traps: the absence of any trapped radical species further supported that no homolytic (radical) processes were involved. The thermal reactions leading to the formation of **5** could not be tested in a similar way with these trapping agents, as the EMPO spin traps were thermally unstable under the elevated temperatures involved.

**Scheme 5:**
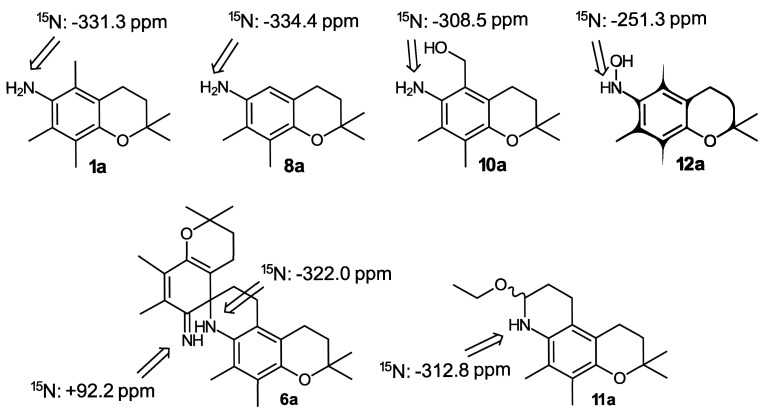
Formulae and ^15^N NMR shifts in the truncated (methyl group instead of isoprenoid side chain, C_16_H_33_), ^15^N-labeled model compounds (**1a**, **8a**, **10a**, **12a**), and the dimerization product (**6a**) and trapping product (**11a**) of the related o-IQM intermediate (cf. also Scheme 1, Scheme 2 and Scheme 3).

Additional structural information about the compounds involved in the generation of o-IQM **5**—beyond the ^1^H and ^13^C NMR data given in the experimental part—was provided by ^15^N NMR, employing ^15^N-labeled model compounds with a methyl group instead of the isoprenoid side chain, which were available from a previous study [34]. The syntheses shown in Scheme 2, Scheme 3 and Scheme 4 were repeated with these model compounds, and the corresponding products were additionally characterized by ^15^N NMR, showing typical shifts in the expected ranges (Scheme 5).

## 3. Conclusions

This study presents unambiguous evidence for the occurrence of the hitherto elusive ortho-iminoquinone methide intermediate (**5**) in the chemistry of α-tocopheramine (**1**). This intermediate represents the counterpart to the well-known ortho-quinone methide **3** derived from α-tocopherol (**2**), as shown in Scheme 1. Three synthesis pathways have been found and optimized to generate o-IQM **5** and convert it further, either by dimerization into its spiro-dimer (**6**) or by trapping with ethyl vinyl ether into the tricyclic product **11**. Both of these follow-up transformations are hetero-Diels–Alder (IEDDA) reactions with an inverse electron. Two approaches generate the o-IQM intermediate **5** by the elimination of water from suitable precursors that carry a hydroxy group either at C-5a (5a-hydroxy-α-tocopheramine, 5-hydroxymethyl-γ-tocopheramine, **10**; see Scheme 2) or at nitrogen (α-tocopherhydroxylamine, **12**; see Scheme 3). The reactions of the latter even provide quantitative yields of both **5** and **11**. The same benefit of quantitative conversion is also true for the carefully optimized direct oxidation of α-tocopheramine (**1**), as shown in Scheme 4, which offers the additional advantage of starting from this readily available compound rather than from one of its derivatives.

Several interesting differences between the chemistries of α-tocopheramine (**1**) and α-tocopherol (**2**)—with regard to the involved ortho-(imino)quinone methide intermediates—have been observed and are summarized in the following list:

- In the α-tocopherol case, the ortho-quinone methide is formed quite readily, with o-QM being a central intermediate in the oxidation chemistry of tocopherol. In contrast, rather special conditions and prerequisites are required for formation of the ortho-iminoquinone methide derived from α-tocopheramine (**1**). Instead of the formation of ortho-quinoid structures, *N*-oxidized compounds are the common oxidation products here.
- In aqueous medium, both α-tocopheramine (**1**) and α-tocopherol (**2**) are oxidized neatly to para-tocopherylquinone (**7**).
- In the absence of water, **2** forms the corresponding *o*-QM **3** almost independently of the oxidant and the solvent used, while the formation of o-IQM **5** from **1** was only observed with special oxidants and in solvents of high ionicity, such as ionic liquids or polar aprotic solvents containing dissolved salts.
- Interestingly, freshly precipitated Ag_2_O, a particularly mild reagent to generate the o-QM quantitatively from α-tocopherol (**2**), is also a good reagent to induce o-IQM formation from α-tocopherhydroxylamine (**10**). While in the former case, it acts as an oxidant that is used in at least equimolar amounts or better in excess, it is a catalyst in the latter case, which cleaves the N-O bond and can be used in substoichiometric amounts (5 mol%). Ag_2_O does not oxidize α-tocopheramine (**1**) directly at all.
- The easy and general formation of 5a-substituted tocopherol via [1,4]-addition to the o-QM intermediate [15,16] has no analog in α-tocopheramine chemistry. The opposite process, the elimination of the 5a-substituent from 5a-substituted α-tocopherols, is a standard reaction in tocopherol chemistry, which always affords the o-QM intermediate. In the tocopheramine realm, the elimination of OH from 5-hydroxy-α-tocopheramine proceeds analogously and affords the o-IQM intermediate. However, it cannot be decided whether this is a general reaction as in the tocopherol case or rather a particular process, because the used 5a-hydroxy-α-tocopheramine is the only 5a-substituted tocopheramine available so far, so no conclusion about the generality of this pathway can be drawn.
- Trapping the o-QM with ethyl vinyl ether is particularly easy; also, in the o-IQM case, the trapping reagent is very suitable. It is best used, in both cases, as a component of solvent mixtures and thus in large excess.
- The ratio of trapping with ethyl vinyl ether and dimerization to the spiro-dimer are different for the intermediate ortho-quinoid structures. While the trapping rate is about 10 times faster than dimerization for the o-QM from α-tocopherol (**2**) [14], the two rates are similar for the o-IQM from α-tocopheramine (**1**).
- The peculiar selectivity of o-QM formation in α-tocopherol (**2**) chemistry, involving C-5a rather than C-7a [15,31], appeared to be similar for α-tocopheramine chemistry. No involvement of C-7a in the formation of o-IQM structures was observed.

## 4. Materials and Methods

All chemicals were of the highest purity available and used without further purification. Bidistilled water was used for all aqueous solutions, extractions, and washing steps. 1,4-Dioxane, *n*-heptane, ethyl acetate, and toluene used in chromatography were distilled before use. The EMIm-Cl ionic liquid was obtained from Nippon Nyukazai Co., Ltd. (Tokyo, Japan).

TLC was performed using Merck silica gel 60 F254 pre-coated plates and flash chromatography on Baker silica gel (40 µm particle size). All products were purified to homogeneity by TLC/GC analysis and satisfied elemental analysis data requirements (±0.3%). Elemental analyses were performed at the Microanalytical Laboratory of the University of Vienna. The melting points are corrected (benzophenone 48–49 °C, benzoic acid 122–123 °C), determined on a Kofler-type micro hot stage with Reichert-Biovar microscope.

UV/Vis spectra were recorded on a LAMBDA 45 UV/Vis spectrophotometer (Perkin Elmer, Waltham, MA, USA) with a range of 400 to 700 nm, scanning speed of 480 nm min^−1^, and quartz glass cuvettes (l = 1.0 cm).

NMR analysis and identification of chromophores: All NMR spectra were recorded on a Bruker Avance II 400 (resonance frequencies 400.13 MHz for ^1^H and 100.63 MHz for ^13^C) equipped with a 5 mm N2-cooled cryoprobe head (Prodigy) with z-gradients at room temperature with standard Bruker pulse programs. The sample was dissolved in 0.6 mL of CDCl_3_ (99.9% D). Chemical shifts are given in ppm, referenced to residual solvent signals. ^1^H NMR data were collected with 32k complex data points and apodised with a Gaussian window function (lb = −0.3 Hz and gb = 0.3 Hz) prior to Fourier transformation. ^13^C spectrum with WALTZ16 1H decoupling was acquired using 64k data points. Signal-to-noise enhancement was achieved by multiplication of the FID with an exponential window function (lb = 1 Hz). All two-dimensional experiments were performed with 1k × 256 data points, while the number of transients (2–16 scans) and the sweep widths were optimized individually. The HSQC experiment was acquired using an adiabatic pulse for the inversion of ^13^C and GARP-sequence for broadband ^13^C-decoupling, optimized for ^1^*J*_(CH)_ = 145 Hz. For the NOESY spectra, a mixing time of 0.8 s was used.

The nomenclature and atom numbering of tocopherols and chromanols as recommended by IUPAC [35,36] was used throughout. ^1^H and ^13^C NMR resonances of the isoprenoid side chain of tocopherols are only insignificantly influenced (Δ < 0.05 ppm) by modifications of the chroman ring [37,38] and are thus listed only once. ^1^H NMR: 1.04–1.50 (m, 21H, 9 × CH_2_ and 3 × CH in isoprenoid side chain), 0.82–0.92 (12H, m, 4 × CH_3_ in isoprenoid side chain); ^13^C NMR: 19.7 (C-4a’), 19.8 (C-8a’), 21.2 (C-2’), 22.7 (C-13’), 22.8 (C-12a’), 24.6 (C-6’), 24.8 (C-10’), 28.0 (C-12’), 32.6 (C-8’), 32.8 (C-4’), 37.3 (C-7’), 37.4 (C-9’), 37.5 (C-5’), 37.5 (C-3’), 39.3 (C-11’), 39.9 (C-1’). In the spiro-dimeric compound **6**, the chroman moiety is conventionally numbered, while “#” denotes the atoms of the spiro-keto moiety. The analytical data [3] and NMR data [39] for α-tocopheramine (**1**) were in agreement with the literature. α-tocopherhydroxylamine (**10**) and the spiro-dimer of α-tocopheramine (**5**) were also available from previous studies [22,32].

### 4.1. Starting Materials

α-tocopheramine (**1**). ^1^H NMR: δ 4.9 (s, br, 2H), 2.66 (2H, m, 4-CH_2_), 2.16 (3H, s, 5a-CH_3_), 2.13 (3H, s, 7a-CH_3_), 2.09 (3H, s, 8b-CH_3_), 1.73 (2H, m, 3-CH_2_), 1.38 (3H, s, 2a-CH_3_). ^13^C NMR: δ 145.0 (C-8a), 134.7 (C-6), 122.3 (C-4a), 120.6 (C-8), 117.8 (C-7), 117.1 (C-5), 74.3 (C-2), 32.7 (C-3), 28.0 (C-2a), 22.7 (C-4), 13.6 (C-5a), 12.6 (C-7a), 11.9 (C-8b). Isoprenoid side chains: see above. Microanalysis: calcd. for C_29_H_51_NO (429.72): C 81.05, H 11.96, N 3.26, found: C 81.23, H 12.10, N 3.21.

γ-tocopheramine (**8**). ^1^H NMR: δ 6.28 (s, 3H, -C-5a), 5.4 (s, br, 2H), 2.64 (2H, m, 4-CH_2_), 2.11 (3H, s, 7a-CH_3_), 2.10 (3H, s, 8b-CH_3_), 1.83 (2H, m, 3-CH_2_), 1.37 (3H, s, 2a-CH_3_). ^13^C NMR: δ 145.0 (C-8a), 136.3 (C-6), 123.8 (C-8), 123.1 (C-4a), 118.2 (C-7), 111.9 (C-5), 74.3 (C-2), 32.5 (C-3), 27.9 (C-2a), 20.8 (C-4), 12.5 (C-7a), 11.8 (C-8b). Isoprenoid side chains: see above. Microanalysis: calcd. for C_28_H_49_NO (415.69): C 80.90, H 11.88, N 3.37, found: C 81.03, H 12.12, N 3.30.

5a-hydroxy-α-tocopheramine, 5-hydroxymethyl-γ-tocopheramine (**10**). ^1^H NMR: δ 6.92 (s, 1H, OH), 4.72 (s, 2H, 5a-CH_2_), 4.40 (s, br, 2H, NH), 2.65 (2H, m, 4-CH_2_), 2.13 (3H, s, 7a-CH_3_), 2.09 (3H, s, 8b-CH_3_), 1.81 (2H, m, 3-CH_2_), 1.38 (3H, s, 2a-CH_3_). ^13^C NMR: δ 147.1 (C-8a), 136.3 (C-6), 123.7 (C-4a), 123.4 (C-7), 121.4 (C-8), 115.3 (C-5), 74.3 (C-2), 67.2 (C-5a), 32.6 (C-3), 28.0 (C-2a), 22.1 (C-4), 13.4 (C-7a), 12.3 (C-8b). Isoprenoid side chains: see above. Microanalysis: calcd. for C_59_H_51_NO_2_ (445.72): C 78.14, H 11.53, N 3.14, found: C 80.86, H 11.98, N 3.52.

α-tocopherhydroxylamine (**12**). ^1^H NMR: δ 4.05 (s, br, 2H, NH, OH), 2.66 (2H, m, 4-CH_2_), 2.24 (3H, s, 5a-CH_3_), 2.12 (3H, s, 7a-CH_3_), 2.10 (3H, s, 8b-CH_3_), 1.83 (2H, m, 3-CH_2_), 1.38 (3H, s, 2a-CH_3_). ^13^C NMR: δ 144.4 (C-8a), 140.3 (C-6), 123.0 (C-4a), 121.7 (C-8), 116.9 (C-7), 116.4 (C-5), 74.3 (C-2), 32.5 (C-3), 28.0 (C-2a), 22.4 (C-4), 13.8 (C-5a), 12.2 (C-7a), 12.0 (C-8b). Isoprenoid side chains: see above. Microanalysis: calcd. For microanalysis: calcd. for C_29_H_51_NO_2_ (445.72): C 78.14, H 11.53, N 3.14, found: C 77.98, H 11.49, N 3.22.

### 4.2. Formation of o-IQM Intermediate ***5***, Dimerization, and Trapping Reactions

Synthesis of trapping product **11** from α-tocopheramine (**1**): α-tocopheramine ([*R*,*R*,*R*] isomer, **1**, 0.430 g, 1.00 mmol) was dissolved in a binary solvent mixture consisting of dimcarb (100 mL) and ethyl vinyl ether (100 mL) under stirring at RT. Neutral aluminum oxide (Brockmann grade 1, 5.00 g, 49 mmol) was added over 5 min under stirring. Urea–hydrogen peroxide complex (0.188 g, 2 mmol) was added in 10 aliquots over 2 h through a dropping funnel. The mixture was stirred for another 2 h. Solids were removed by filtration, washed with dimcarb (twice 50 mL). The organic phases were combined and the solvent removed in a rotavaporator at 80 °C to afford **11** as a waxy, colorless solid (0.502 g, quant.).

Synthesis of trapping product **11** from 5a-hydroxy-α-tocopheramine (**10**): 5a-hydroxy-α-tocopheramine (5-hydroxymethyl-γ-tocopheramine, [*R*,*R*,*R*] isomer, **10**, 0.445 g, 1.00 mmol) was dissolved in o-dichlorobenzene (50 mL) and ethyl vinyl (40 mL) ether under stirring at RT in a stainless-steel pressure reactor (volume 120 mL). The vessel was closed and heated to 120 °C under stirring for 2 h. After cooling, the vessel was opened, the solids were removed by filtration, and the solvents removed in vacuo. The oily residue was chromatographed on silica gel (n-heptane/toluene (*v*/*v* = 9/1) to give trapping product **11** as a waxy, colorless solid (0.236 g, 47%). The reaction proceeded similarly with the solvents DMSO (44% yield) and diphenyl ether (23% yield).

Synthesis of trapping product **11** from α-tocopherhydroxylamine (**8**): α-tocopheramine ([*R*,*R*,*R*] isomer, **1**, 0.430 g, 1.00 mmol) was dissolved in ethyl vinyl ether (50 mL) under stirring at RT. Freshly precipitated and dried silver oxide (0.012 g, 5 mol% rel. to **1**) was added at once and the mixture was stirred for 2 h. Solids were filtered off and the solvent was removed by rotavaporation. The residue consisted of neat (GC) trapping product **11** as a waxy, colorless solid (0.499, quant.).

(2*R*)-2-[(4*R*,8*R*)-4,8,12-Trimethyltridecyl]-7-ethoxy-2,9,10-trimethyl-3,4,5,6,7,8-hexahydro-2*H*-1-oxa-8-azaphenanthrene is the trapping product of o-IQM **5** with ethyl vinyl ether (**11**). Atom numbering (A-D) refers to the former ethyl vinyl ether moiety: C_A_H_3_-C_B_H_2_ – C_C_H = C_D_H_2_. ^1^H NMR: δ 5.65 (dd, 1H, C_C_H), 3.62 (q, 2H, C_B_H_2_), 3.20 (s, br, 1H, NH), 2.62–2.68 (2H, 5a-CH_2_), 2.64 (2H, m, 4-CH_2_), 2.40–2.32 (m, 1H, C_D_H_2_), 2.14 (3H, s, 7a-CH_3_), 2.11 (3H, s, 8b-CH_3_), 1.98–1.92 (m, 1H, C_D_H_2_), 1.82 (2H, m, 3-CH_2_), 1.37 (3H, s, 2a-CH_3_), 1.24 (t, 3H, C_A_H_3_). ^13^C NMR: δ 143.9 (C-8a), 132.5 (C-6), 122.8 (C-4a), 120.3 (C-8), 118.0 (C-7), 117.4 (C-5), 78.3 (C_C_H), 74.2 (C-2), 61.5 (C_B_H_2_), 32.5 (C-3), 28.1 (C-2a), 26.2 (C_D_H_2_), 22.7 (C-4), 20.9 (C-5a), 15.4 (C_A_H_3_), 12.8 (C-7a), 12.2 (C-8b). Isoprenoid side chains: see above. Microanalysis: calcd. for C_33_H_57_NO_2_ (499.81): C 79.30, H 11.50, N 2.80, found: C 79.14, H 11.24, N 2.92.

Synthesis of spiro-dimer **5** from α-tocopheramine (**1**): α-tocopheramine ([*R*,*R*,*R*] isomer, **1**, 0.430 g, 1.00 mmol) was dissolved in dimcarb (200 mL) under stirring at RT. Neutral aluminum oxide (Brockmann grade 1, 5.00 g, 49 mmol) and urea–hydrogen peroxide complex (0.188 g, 2 mmol) were mixed as solids, and the fine powder was added through a dropping funnel in one portion. The mixture was stirred for 2 h. Solids were removed by filtration and washed with dimcarb (twice 100 mL). The dimcarb phases were combined and heated in a rotavaporator at 80 °C to afford **5** as a waxy, colorless solid (0.43 g, quant.). Lower-yield syntheses starting from either α-tocopherhydroxylamine (**8**) or 5a-hydroxy-α-tocopheramine (**10**) are not given here, as the above protocol allows for synthesis in a quantitative yield without the need of chromatographic purification [32].

Spiro-dimer of α-tocopheramine (**5**): ^1^H NMR: δ 2.62 (2H, m, 4-CH_2_), 2.45 (2H, m, 4#-CH_2_), 2.24 (3H, s, 5a-CH_3_), 2.16 (3H, s, 5a#-CH_3_), 2.05 (3H, s, 7a-CH_3_), 1.83 (3H, s, 7a#-CH_3_), 2.04 (3H, s, 8b-CH_3_), 1.96 (3H, s, 8b#-CH_3_), 1.60–1.64 (4H, m, 3-CH_2_ and 3#-CH_2_), 1.04–1.50 (m, 42H, 18×CH_2_ and 6×CH in two isoprenoid side chains), 1.22 (3H, s, 2a-CH_3_), 1.21 (3H, s, 2a#-CH_3_), 0.82–0.92 (24H, m, 4a-CH_3_, 8a-CH_3_, 12a-CH_3_, 13-CH_3_, 4a#-CH_3_, 8a#-CH_3_, 12a#-CH_3_, 13#-CH_3_). ^13^C NMR: δ 145.3 (C-8a), 142.4 (C-8a#), 122.9 (C-8), 122.5 (C-8#), 10.8 (C-8b), 123.0 (C-8b#), 122.8 (C-7), 125.4 (C-7#), 11.0 (C-7a), 10.6 (C-7a#), 140.3 (C-6), 154.9 (C-6#), 112.5 (C-5), 68.7 (C-5#), 18.9 (C-5a), 25.8 (C-5a), 116.2 (C-4a), 116.1 (C-4a#), 19.0 (C-4), 16.5 (C-4#), 30.4 (C-3#), 30.2 (C-3), 73.7 (C-2#), 75.0 (C-2), 22.3 (C-2a, C-2a#). Isoprenoid side chains: see above. Microanalysis: calcd. for C_58_H_98_N_2_O_2_ (855.41): C 81.44, H 11.55, N 3.27, found: C 81.30, H 11.54, N 3.08.

## Data Availability

The original contributions presented in this study are included in the article. Further inquiries can be directed to the corresponding author.
